# Patterns of Reading Performance in Acute Stroke: A Descriptive Analysis

**DOI:** 10.3233/BEN-2009-0258

**Published:** 2010-06-10

**Authors:** Lauren L. Cloutman, Melissa Newhart, Cameron L. Davis, Vijay C. Kannan, Argye E. Hillis

**Affiliations:** ^1^Department of NeurologyJohns Hopkins University School of MedicineBaltimoreMDUSA; ^2^Department of Physical Medicine and RehabilitationBaltimoreMDUSA; ^3^Department of Cognitive ScienceJohns Hopkins UniversityBaltimoreMDUSA

**Keywords:** Dyslexia, oral reading, acute stroke

## Abstract

One of the main sources of information regarding the underlying processes involved in both normal and impaired reading has been the study of reading deficits that occur as a result of brain damage. However, patterns of reading deficits found acutely after brain injury have been little explored. The observed patterns of performance in chronic stroke patients might reflect reorganization of the cognitive processes underlying reading or development of compensatory strategies that are not normally used to read. Method: 112 acute left hemisphere stroke patients were administered a task of oral reading of words and pseudowords within 1–2 days of hospital admission; performance was examined for error rate and type, and compared to that on tasks involving visual lexical decision, visual/auditory comprehension, and naming. Results: Several distinct patterns of performance were identified. Although similarities were found between the patterns of reading performance observed acutely and the classical acquired dyslexias generally identified more chronically, some notable differences were observed. Of interest was the finding that no patient produced any pure semantic errors in reading, despite finding such errors in comprehension and naming.

## 1. Introduction

One of the main sources of evidence for the underlying processes involved in both normal and impaired reading has been the study of individuals who have developed reading deficits as a result of brain damage. From the patterns of preservation and impairment in the reading (and other task) performance of these individuals, inferences have been drawn regarding the nature of the underlying processes involved, and how these may be differentially disrupted following brain damage.

Studies have identified several commonly occurring patterns of performance, which have traditionally been viewed as reflecting specific dyslexic syndromes (see [11,26] for reviews). Although these syndromes are heterogeneous, with inherent variability in the range and severity of deficits observed within each group, their classification reflects key differences in relation to word and nonword reading ability.

One of the most widely studied of these classic dyslexic syndromes is deep dyslexia, which is associated with deficits in the reading of both words and nonwords. The central defining feature of the syndrome is the presence of semantic errors (e.g., cat → dog), in oral word reading (e.g. [1–3,9,17,30,31,38,39,47, 49,54,55]). Other key features include the production of visual (e.g., mouse → house), and morphological (e.g., import → imported) errors in word reading, coupled with a severe difficulty (or complete inability) to read nonwords. In addition, reading accuracy in deep dyslexia is often influenced by the effects of concreteness (with concrete words read more accurately than

ISSN 0953-4180/09,10/$17.50 © 2009/2010 – IOS Press and the authors. All rights reserved abstract words), and grammatical class (with nouns read more accurately than other syntactic classes; see e.g. [30,36]).

A second commonly observed reading pattern is that of phonological dyslexia, which involves a comparative inability to read nonwords compared to real words (e.g. [28,30,49,50,53,58,62]). Predominant errors in nonword oral reading include orthographically/phonologically related lexicalization errors (e.g., fown → frown), and phonological errors. In addition, similar to deep dyslexic individuals, phonological dyslexic individuals have been observed to make morphological and visual errors in real word reading, as well as demonstrating effects of concreteness and grammatical class on oral reading accuracy [30]. In fact, the patterns of reading performance in deep and phonological dyslexias are so similar (with the presence of semantic errors in deep dyslexia the only distinguishing feature), a number of researchers have argued that phonological and deep dyslexia are not separate disorders but are two ends of a continuum of reading performance, with deep dyslexia representing an extreme form of phonological dyslexia [21,30,56].

A third identified pattern of reading is that of surface dyslexia, which is characterized by a selective word reading deficit involving regularization errors when attempting to read words which violate spelling-sound correspondences (e.g., 'pint' pronounced as rhyming with 'mint' [6,10]). Words with regular spelling-sound correspondences, and nonwords are read relatively accurately.

Finally, there are a number of individuals who are almost completely unable to read aloud either words or nonwords, or only do so very slowly and laboriously in a letter-by-letter fashion, in the syndrome known as (pure) alexia (e.g. [12,18,24,27,32,48]). When words are able to be read letter-by-letter by these dyslexic individuals, errors generally bear a visual similarity to the target.

Based on these different patterns of reading performance observed in acquired dyslexia, researchers have proposed models of both normal and impaired oral reading [7,14,15,23,29,44,46,51,52]. For example, the dissociation between dyslexic individuals who can read nonwords but not irregular words (surface dyslexia), and those who can read at least some words but not nonwords (phonological dyslexia), has been taken as evidence for the existence of two independent processes involved in reading [44–46]. The first process, known as the sublexical or assembled route, involves the use of known grapheme-phoneme conversion (GPC) rules to derive the correct phonological representation of the letter string. This route is capable of reading words that conform to regular spelling-sound correspondences, as well as nonwords. The second route, known as the lexical or addressed route, involves accessing the phonological form of the word from orthography via the word's semantic representation. This mechanism makes it possible to read all familiar words (with both regular and irregular pronunciations), but cannot read unfamiliar words or nonwords. In addition to these two routes, a third, direct route has sometimes been proposed, in which a phonological lexical representation is accessed from an orthographic lexical representation directly, without the need for semantic mediation (e.g. [46,59]; but see [4]). Although models may differ in relation to their architecture and processing assumptions (see e.g. [44] versus [16]), there is some agreement that these lexical (whole-word) and sublexical reading processes are involved in the translation from orthography to phonology. However, other accounts of reading posit only a single mechanism of reading, involving computation of an output via parallel processing of distributed orthographic and phonological (and in some models, semantic), representations based on statistical relationships between input and output (e.g. [7]).

According to dual-route (or dual-process) models, phonological and deep dyslexia result from impairments to the sublexical route, resulting in a difficulty with reading nonwords (and unfamiliar words), while surface dyslexia is attributed to an impaired lexical route, meaning that reading can only occur via grapheme-phoneme correspondence [44]. The additional semantic errors which define deep dyslexia are argued to result from additional impairments either to the semantic system itself, or its ability to activate the word's phonological representation. Although it is often assumed that deep dyslexia reflects a combination of a deficit in semantics and a deficit in sublexical processes, it should be noted that this pattern of performance can also arise from an impairment in access to semantics specifically from vision or access to phonological representation from intact semantics, combined with impaired sublexical processes [5].

However, the patterns of performance on which these models of normal and impaired reading are based come almost exclusively from the study of patients with subacute or chronic lesions. Although there are studies that have examined acquired dyslexia within two or three weeks after brain damage (e.g. [25,55]), the vast majority of studies have examined dyslexia months (e.g. [27,

31,42,48]), or years (e.g. [22,35,40,57,60]), post-onset. Very few, if any, have examined patterns of impaired reading performance observed after brain damage in the acute phase (i.e., within one to two days of brain damage). A successful model of normal and impaired reading performance must be able to account not only for patterns of reading deficits found after chronic brain damage, but also those found acutely. Patterns of reading performance that are found only after the acute phase would be likely to represent some sort of reorganization of the cognitive system, or reliance on compensatory mechanisms or brain regions that are not normally used for reading. The current study aimed to identify the patterns of reading performance found in the acute stage of brain injury by examining oral reading in a large sample of acute stroke patients.

## 2. Method

### *2.1. Participants*

A series of 331 right-handed patients with acute ischemic stroke, were initially enrolled upon meeting the following inclusion criteria: premorbid proficiency in English, no known hearing loss or uncorrected visual impairment; no history of dementia, previous symptomatic stroke, or other neurological disease; and no hemorrhage on initial scans. Testing was attempted to be completed within 24 hours of stroke onset; however, some patients were included who were tested between 24 and 48 hours of stroke onset (usually because they were admitted close to or after 24 hours after initial symptoms). Following enrollment, 197 patients were excluded from the current study due to: right hemisphere or bilateral infarcts, the absence of infarct on MRI or CT scans, a history of previous ischemic stroke, and/or less than a 10th grade level of education. Of the remaining 135 patients, 23 failed to complete the oral reading task, and were removed from the study. For the final group of 112 patients, the mean age was 59.4 ± 15.03 standard deviation (SD) years; the mean education level was 13.3 ± SD 2.3 years.

### *2.2. Language Tasks*

#### *2.2.1. Reading Tasks*

Within 48 hours of stroke onset participants were presented with a 58 item oral reading task, involving both word and nonword reading. For the majority of patients, the task involved reading 34 words and 24 nonwords (however, for a small subset of patients, the task involved reading 36/22 or 35/23 words/nonwords, due to an error in the stimuli). Words ranged in length from three to seven letters (mean = 5 ± SD 1.3 letters), while nonwords tended to be slightly shorter, ranging from three to five letters (mean = 4.1 ± SD 0.7 letters) because they were created by changing one letter of 24 words 3–5 letters in length, matched in frequency to the 24 3–5 letter words on the word reading list.

In addition to the oral reading task, patients performed a visual lexical decision task on the same items they read aloud; and a reading comprehension task comprised of a 17 item visual word/picture verification task, in which each of 17 items was presented three times (once every 17 items): once with a semantically related foil; once with a phonologically related foil; and once with the target, in counterbalanced order across three presentations of the same set of items. The patient was required to accept the target word and reject both foils to receive credit for the item ([19], for the validity of this task in the auditory modality).

#### *2.2.2. Additional Tasks*

In addition to the reading tasks, patients were also given an auditory comprehension task, comprised of an auditory variant of the word/picture verification task described above (although with a different set of stimuli); and a 17 or 30 item untimed picture naming task, with line drawings taken from [33].

### *2.3. Data analysis*

On all tasks, the initial response was scored. Oral reading performance was coded for word/nonword error rate and type. Error categories for word reading included: 1) semantic errors, which included coordinates (e.g. "cat" for the target word 'dog'), associates (e.g., bone for dog), superordinates (e.g., animal for dog), subordinates (e.g., Labrador for dog), and circumlocutions (e.g., they bark a lot); 2) phonological/visual errors, which included visual (words related to the target with at least two-thirds of letters/phonemes in the same position, e.g., dot for dog), phonemic (nonwords related to the target by initial or final phoneme, e.g., dop for dog), neologisms (nonwords phonologically unrelated to the target, e.g., mip for dog), phonologically plausible (e.g., pronouncing the 'o' in dog as in the word 'doe'), and morphological (e.g., dogs for dog); 3) mixed errors, which bore both a semantic and phonological/visual relationship to the target (e.g., frog for dog); 4) unrelated (e.g., table for dog); 5) no responses; and 6) other errors, which could not be categorized. The error categories for nonword reading were the same as those for word reading, with the exclusion of semantic, mixed, and phonologically plausible errors (which were not possible for nonwords). These error categories were also used in the scoring/categorization of oral picture naming performance.

Based on the error types observed for words and nonwords, patients were categorized into five groups, based on the patterns of errors: 1) No Error – patients who made no errors in reading; 2) Phonological/Visual Only – patients who made only phonological/visual errors in word/nonword reading; 3) Nonword Deficit – patients who made errors predominantly on nonwords, classified as at least twice as many errors on nonwords than words (some of whom might meet criteria for phonological dyslexia); 4) Word Deficit – patients who made errors predominantly on words, classified as at least twice as many errors on words than nonwords (some of whom might meet criteria for surface dyslexia); and 5) General Oral Reading Deficit – patients who made errors on both words and nonwords. We did not classify them according to the dyslexia syndromes described earlier (phonological, surface, and deep dyslexia) for two reasons. Firstly, the current study was designed to be an examination of general word and nonword reading performance, and as such, the stimuli were not designed to measure all aspects identified in the aforementioned research with chronic patients (e.g., concreteness and grammatical class effects). Secondly, many patients with acquired dyslexia in the chronic phase also fail to meet criteria for these syndromes.

## 3. Results

Although all patients completed the oral reading task, which was of most interest in the current study, not all patients completed the remaining lexical decision, reading comprehension, oral picture naming, and auditory comprehension tasks. Where the numbers of patients completing the task did not match the total number of patients for the group, patient numbers for the task are stated or indicated in squared parentheses.

### *3.1. Whole group analysis*

Of the 112 patients who completed the oral reading task, 92 (82%) produced at least one error (Table 1). Although the group as a whole had an overall error rate of around 18%, there was a great deal of variability in reading performance, ranging from individuals who made no errors, to those who could not read any of the word/nonword stimuli presented. As can be seen in Table 1, a greater number of errors were produced on nonwords than words, with phonological errors (particularly visual and phonemic errors) the predominant error type for both. In addition to oral reading, deficits were also observed in the other reading tasks examined. Of the 102 patients who completed the visual lexical decision task, 88% (90 patients) demonstrated some degree of error, producing a greater number of errors on nonwords compared to words. A fewer number of patients demonstrated deficits in reading comprehension [109 patients], with just over half the group (51%) producing errors on this task, with a roughly equivalent number of semantic and phonological errors. Deficits were also observed in oral picture naming [107 patients], with just over 70% of patients producing errors, and auditory comprehension [107 patients], with 50% of the patients who completed this task demonstrating some degree of error.

No patient produced a pure semantic error on the oral reading task, although five patients made mixed errors (1 Word Only, 1 Phonological/Visual Only, and 3 General Oral Reading Deficit). However, the rates of these mixed errors were low – less than 1% error rate for the group as a whole, and 3–6% for the individuals who made mixed errors (1–2 mixed errors each). In contrast to the absence of pure semantic errors in oral reading, these errors were observed in oral picture naming (7% error rate), reading comprehension (9% error rate), and auditory comprehension (10% error rate). Of the 85 patients who fully completed all four tasks, 21% made semantic errors on all tasks excluding oral reading (even mixed errors in oral reading were not observed in these patients). In fact, seven patients (1 No Error, 2 General Oral Reading Deficit, and 4 Phonological/Visual Only), were found to produce semantic errors at a rate of greater than 10% of items across the other three tasks (naming, reading/auditory comprehension), but failed to make a single semantic (or mixed) error in oral reading.

### *3.2. Specific group analysis*

The 112 patients who completed the reading task were divided into five groups based on their oral reading profiles: No Error, Phonological/Visual Only, Word Deficit, Nonword Deficit, and General Oral Reading Deficit (Table 1).

Table 1
Error rates (percentage of total responses) for all patients in study, as well as specific reading groups

| | All | | NE | | VO | | WD | | NWD | | GRD | |
|---|---|---|---|---|---|---|---|---|---|---|---|---|
| | Mean | SD | Mean | SD | Mean | SD | Mean | SD | Mean | SD | Mean | SD |
| **Oral Reading** | | | | | | | | | | | | |
| Total Errors | 18.0 | 22.8 | 0 | 0 | 4.0 | 3.2 | 1.7 | 0 | 21.2 | 14.8 | 45.4 | 34.0 |
| *Words* | | | | | | | | | | | | |
| Total Errors | 11.1 | 20.2 | – | – | 1.0 | 1.4 | 1.7 | 0 | 8.3 | 9.1 | 40.9 | 32.8 |
| *Error Type:* | | | | | | | | | | | | |
| Semantic | 0 | 0 | – | – | – | – | 0 | 0 | 0 | 0 | 0 | 0 |
| Mixed | 0.2 | 0.8 | – | – | – | – | 0.6 | 1.3 | 0.1 | 0.4 | 0.6 | 1.5 |
| Phonological | 7.8 | 13.9 | – | – | – | – | 1.8 | 1.6 | 6.3 | 6.7 | 27.3 | 23.2 |
| Visual | 3.0 | 4.5 | – | – | 1.0 | 1.4 | 0 | 0 | 3.2 | 4.0 | 8.4 | 5.8 |
| Phonemic | 3.1 | 6.1 | – | – | – | – | 1.2 | 1.6 | 2.5 | 3.1 | 10.9 | 10.8 |
| Neologism | 1.0 | 7.2 | – | – | – | – | 0 | 0 | 0.1 | 3.1 | 6.0 | 16.9 |
| Plausible | 0.2 | 0.8 | – | – | – | – | 0 | 0 | 0.2 | 0.8 | 0.5 | 1.1 |
| Morphological | 0.5 | 1.7 | – | – | – | – | 0.6 | 1.3 | 0.4 | 1.2 | 1.5 | 3.4 |
| Unrelated | 0.6 | 2.5 | – | – | – | – | 0 | 0 | 0.3 | 1.4 | 2.8 | 5.1 |
| No Response | 2.2 | 10.2 | – | – | – | – | 0 | 0 | 1.2 | 4.0 | 9.8 | 22.8 |
| Other | 0.3 | 1.2 | – | – | – | – | 0.6 | 1.3 | 0.3 | 1.3 | 0.6 | 1.9 |
| *Nonwords* | | | | | | | | | | | | |
| Total Errors | 27.9 | 30.2 | – | – | 7.6 | 6.3 | 0 | 0 | 39.5 | 25.2 | 51.6 | 37.1 |
| *Error Type:* | | | | | | | | | | | | |
| Phonological | 21.3 | 22.3 | – | – | – | – | 0 | 0 | 31.9 | 17.9 | 32.7 | 29.4 |
| Visual | 14.2 | 15.0 | – | – | 7.6 | 6.3 | 0 | 0 | 23.4 | 15.1 | 14.2 | 13.1 |
| Phonemic | 5.7 | 7.6 | – | – | – | – | 0 | 0 | 8.3 | 5.9 | 11.2 | 11.5 |
| Neologism | 1.4 | 8.2 | – | – | – | – | 0 | 0 | 0.2 | 1.3 | 7.4 | 19.1 |
| Unrelated | 0.6 | 2.6 | – | – | – | – | 0 | 0 | 0.3 | 1.1 | 2.4 | 5.8 |
| No Response | 5.2 | 15.3 | – | – | – | – | 0 | 0 | 5.6 | 14.4 | 15.4 | 26.3 |
| Other | 1.0 | 3.0 | – | – | – | – | 0 | 0 | 1.6 | 3.8 | 1.3 | 3.1 |
| **Other Tasks** | | | | | | | | | | | | |
| *Lexical Decision* | | | | | | | | | | | | |
| Total Errors | 11.7 | 10.5 | 4.3 | 6.7 | 12.1 | 11.7 | 3.8 | 3.4 | 13.4 | 9.7 | 18.7 | 11.3 |
| Words | 4.9 | 8.2 | 7.5 | 13.1 | 1.6 | 2.2 | 5.8 | 7.0 | 5.4 | 8.3 | 11.7 | 12.4 |
| Nonwords | 21.4 | 22.7 | 4.3 | 6.7 | 27.0 | 29.1 | 2.4 | 2.5 | 24.6 | 20.0 | 29.4 | 28.9 |
| *Reading Comprehension* | | | | | | | | | | | | |
| Total Errors | 14.2 | 21.7 | 8.7 | 23.4 | 17.0 | 26.6 | 1.2 | 2.6 | 14.8 | 21.1 | 19.3 | 18.4 |
| Semantic | 8.9 | 18.2 | 7.5 | 23.6 | 11.1 | 22.6 | 0 | 0 | 8.9 | 16.6 | 10.9 | 13.0 |
| Phonological | 7.7 | 15.6 | 6.5 | 23.6 | 10.0 | 15.1 | 1.2 | 2.6 | 7.9 | 14.5 | 8.0 | 8.8 |
| *Oral Picture Naming* | | | | | | | | | | | | |
| Total Errors | 21.3 | 27.4 | 11.6 | 16.4 | 17.8 | 26.6 | 0 | 0 | 18.2 | 23.9 | 48.2 | 31.7 |
| Semantic | 6.6 | 7.5 | 6.4 | 10.0 | 1.1 | 3.2 | 0 | 0 | 2.9 | 7.5 | 5.0 | 5.3 |
| Phonological | 4.7 | 12.3 | 2.1 | 7.6 | 4.4 | 6.4 | 0 | 0 | 6.6 | 7.1 | 16.2 | 22.3 |
| *Auditory Comprehension* | | | | | | | | | | | | |
| Total Errors | 11.7 | 19.9 | 2.1 | 4.8 | 12.1 | 20.8 | 1.2 | 2.6 | 12.1 | 19.3 | 25.4 | 26.5 |
| Semantic | 10.3 | 18.7 | 1.8 | 3.9 | 10.3 | 17.3 | 1.2 | 2.6 | 10.5 | 18.2 | 21.4 | 26.8 |
| Phonological | 5.1 | 12.2 | 1.8 | 4.3 | 3.3 | 7.7 | 0 | 0 | 5.1 | 9.0 | 11.1 | 23.3 |

*Note.* All = all acute patients in study; NE = No Error group; VO = Visual Only group; WD = Word Deficit group; NWD = Nonword Deficit group; GRD = General Oral Reading Deficit group.

### 3.2.1. *No error*

Of the 112 patients, 18% (20 patients) produced no errors in oral reading. However, although this group demonstrated preserved oral reading, 70% made errors in at least one of the other reading tasks examined. Interestingly, 60% of the patients in this group produced errors in the visual lexical decision task (which involved the same words as the oral reading task), although error rates were generally low. In addition, 26% of those who those completed the reading comprehension task [19 patients] showed some degree of deficit. In relation to the non-reading tasks, errors were also observed on oral picture naming [19 patients], with 58% demonstrating some degree of error, and auditory comprehension (20% of the patients in the group), although again, error rates were not high for this task.

### 3.2.2. *Phonological/Visual only*

Of the 112 patients, 15% (17 patients) produced only phonological/visual errors. The majority of patients in this group (70%) made errors on nonwords only (usually reading them as words), and as such, the error rates for nonwords, was much higher than those of words. All the patients in this group who completed the lexical decision task [15 patients] made errors on the task, with errors predominantly on nonwords, compared to words, fitting the pattern seen in their oral reading of the misidentification of nonwords as words. In addition to errors on lexical decision, approximately half of the group (53%) produced errors on the reading comprehension task, with similar rates of errors on both semantic and phonological foils. In relation to performance on the other tasks examined, for the patients who completed the tasks [16 patients for each task], 69% produced errors on oral naming, while 56% were found to demonstrate some degree of error on auditory comprehension, with the error rates on both tasks comparable to those found for the group as a whole.

*3.2.3. Word deficit*

Of the 112 patients, only 4% (5 patients) produced errors on predominantly words. A predominance of word errors only occurred in patients who produced no nonword errors at all. There were few errors produced by this group, but the majority were visual/phonological in nature, and none were phonologically plausible. Therefore, despite their relatively preserved reading of nonwords, they could not be described as having surface dyslexia. As with oral reading, error rates on the other reading tasks examined were comparatively low for both lexical decision (4 patients, or 80% of the group, producing task errors), and reading comprehension (1 patient, or 20% of the group). Interestingly, in line with their oral reading, and in contrast to the other groups examined, these patients made more lexical decision errors on words (6%) than nonwords (2%). In the non-reading tasks, only one patient produced an error in auditory comprehension, and interestingly, no patient demonstrated any oral naming errors. Thus, it appears the overall deficits observed by these patients were milder than the other patient groups examined (at least for the tasks in the current study). However, this group was small (only 5 patients), so caution must be taken in interpreting any possible patterns of performance.

*3.2.4. Nonword deficit*

Of the 112 patients, 46% (51 patients) produced errors predominantly on nonwords, compared to words. Errors in this group were predominantly phonological in nature, with the majority of these phonological errors bearing a visual relationship to the target. In relation to the other reading tasks examined, of the patients who completed the task [47 patients], 96% demonstrated some degree of error on the lexical decision task, producing a much higher rate of errors on nonwords compared to words. In addition, 60% of the patients who completed the reading comprehension task [50 patients] demonstrated errors on this task, with comparable rates of semantic and phonological errors. In relation to the non-reading tasks examined, 75% of the patients who completed the oral picture naming task [48 patients] produced errors, producing more phonological than semantic errors, while 51% of the patients who completed the auditory comprehension task [47 patients] produced errors, with a greater proportion of semantic than phonological errors.

*3.2.5. General oral reading deficit*

Of the 112 patients, 17% (19 patients) produced errors on both words and nonwords at comparable rates. Not surprisingly, this group was found to have the worst overall reading performance of all the groups examined (46% error rate). As with the other groups, errors were predominantly phonological/visual in nature for both words and nonwords. A high rate of no responses was also produced for both words and nonwords. In relation to the other reading tasks examined, this group also produced a higher rate of errors in lexical decision [15 patients; 93% producing errors] and reading comprehension [18 patients; 67% producing errors] than the other groups examined. In addition, deficits in the non-reading tasks were found to be much higher than the other groups examined for both oral picture naming (95% producing errors), and auditory comprehension (89% producing errors).

## 4. General discussion

The current study aimed to examine the oral reading performance of a large sample of patients with acute stroke, in an attempt to identify what patterns of reading deficit, if any, are observed in the acute stages of brain injury. While there were a number of patients who produced no errors on the oral reading task, the majority of patients (over 80%) demonstrated some degree of impairment, and number of distinct patterns of performance were identified.

The vast majority of patients who demonstrated deficits on the current reading task were found to have a comparatively greater difficulty with the oral reading of nonwords compared to words, with almost half the patients examined showing this pattern of reading performance. In addition, a number of patients displayed a general reading deficit, demonstrating difficulties with the oral reading of both words and nonwords. Not surprisingly, this group was found to have the greatest severity of impairment of all the reading deficit groups identified. The majority of errors produced by both the nonword and general reading deficit groups were phonological in nature, with visual and phonemic errors predominating in both word and nonword reading. Another group was also found to produce only visual errors in word and nonword reading, with the majority of their errors occurring for nonword reading (i.e., lexicalization errors), although error rates were not high (approximately 1% and 8% for words and nonwords respectively). Finally, patients who produced deficits on words in the presence of intact nonword reading were rare, and their deficits were comparatively mild compared to the other groups identified, especially those who produced nonword errors in addition to word errors.

Although direct comparisons between the current acute reading patterns and the dyslexic syndromes found more chronically cannot be made, we can attempt identify potential similarities and differences in word/nonword reading patterns, and types of errors produced. As noted in the introduction, phonological dyslexia is associated with a comparative inability to read nonwords compared to real words (e.g. [30,50, 58]), with production of predominantly visually related lexicalization errors, and phonological errors in nonword reading. This pattern of reading performance is very similar to that found in the current study for the nonword reading group. The visual/phonological errors only group also produced predominantly lexicalization errors on nonword reading. However, this group produced no other phonological error types, and their error rates were generally much lower than that found for the nonword group (see Table 1).

According to dual-route models (based on chronic reading deficits), phonological dyslexia result from impairments to the sublexical route, meaning that successful reading can only be achieved via the (comparatively intact) lexical route [44]. As this pattern was relatively common, a reliance on the lexical route (so-called 'semantic' reading), may be a dominant compensatory strategy for reading following acute brain injury. Alternatively, the sublexical route may simply be more susceptible to damage, either as a result of the neuroanatomical location of the brain regions involved, or due to the fact that translation from orthography to phonology is one of the later learned cognitive functions, generally not being acquired until school-age. Those who also produced a high rate of word errors in addition to nonword errors (the general reading deficit group), may have had an additional impairment to the lexical route as well as the sublexical route, resulting in impaired performance on both word and nonword reading, although damage to other associated reading processes, such as visual processing, cannot be ruled out.

In contrast, although there were a small group of patients who demonstrated good nonword reading in the face of a (mild) difficulty in word reading, no patient demonstrated the pattern similar to that generally attributed to surface dyslexia, that is, phonologically plausible errors in irregular word reading coupled with intact regular word and nonword reading. In fact, very few individuals in the group as a whole produced any phonologically plausible errors, and those who did also tended to produce errors in nonword reading. Surface dyslexia has been suggested to reflect a reliance on sublexical reading mechanisms, in the presence of impaired semantics and/or impaired access to phonological representations from intact semantics [37]. The failure to observe such a pattern in the current study raises the possibility that reliance purely on a sublexical process is a learned compensatory strategy for reading. However, it is difficult to determine the significance of the absence of an acute reading pattern similar to surface dyslexia due to the lack of prevalence rates for the specific acquired dyslexic syndromes. It is possible that acquired surface dyslexia is a particularly rare reading deficit, accounting for the failure to find a patient in the current study who fitted this profile, despite the relatively large number of patients examined.

Of interest in the current study is the finding that no patient tested within 48 hours of stroke produced pure semantic errors acutely (the defining feature of the chronic syndrome of deep dyslexia), despite finding a high rate of such errors in oral picture naming. A small number of patients did produce mixed reading errors, which bore both a semantic and phonological relationship to the target. However, with this type of error, it is difficult to determine whether there is a semantic contribution to the errors produced, or if the errors are purely phonological and bear a semantic relationship to the target by chance – a possibility that cannot be ruled out given the high rate of phonological errors produced by the patients examined. As with surface dyslexia, it is possible that we failed to observe a pattern similar to deep dyslexia in the current study due to the potential rarity of the reading deficit. However, it is interesting that no semantic errors were observed in the oral reading task despite the high prevalence of such errors on other tasks assessed, that is, oral naming, and auditory/visual comprehension.

It is feasible that the current finding of a lack of semantic errors in naming acutely is evidence for the possibility that such errors do not occur during the acute stage of brain injury. The majority of previous studies that have examined semantic errors in acquired dyslexia have studied deep dyslexic patients months (e.g. [31, 41]), or years (e.g. [3,54]), after their initial brain injury. One of the earliest timeframes in which deep dyslexia has been reported is 3 weeks post infarct [55]. So, it is possible that the current findings reflect that the production of semantic errors in oral reading may not occur acutely, and may take several days or weeks to be observed. If this is indeed the case, then it would suggest that these errors, and therefore the pattern of reading known as deep dyslexia, may reflect compensation or reorganization following brain damage. But what could be the possible reason for this?

One possibility is the proposal that deep dyslexia reflects right hemisphere reading [20,43,55]. The right hemisphere is likely always to be engaged in some respects in reading, as indicated by functional imaging studies that show activation of right (albeit less than left) hemisphere areas during reading by right-handed individuals [8,34,61]. However, normally the right hemisphere is not used for reading independently of the left hemisphere. It has been proposed that the right hemisphere codes semantics, or word meanings, more coarsely or broadly than the left hemisphere, such that semantically related words (e.g. car and truck) might not have distinct semantic representations in the right hemisphere [24,42]. Reliance on right hemisphere semantic representations for reading, in the absence of left hemisphere more fine grained semantic representations, would plausibly lead to the production of semantic errors in reading. It is plausible that reliance on right hemisphere mechanisms of reading would not occur acutely, since functional imaging studies in acute aphasic individuals do not show the increase in activation of the right hemisphere that is often seen later in stroke in the same individuals [13].

The task remains to determine whether or not deep dyslexia is indeed only a subacute/chronic disorder, a task made difficult by the fact that a failure to observe the deficit acutely does not equate to proof that it does not occur. More compelling evidence that such errors only occur outside of the acute phase, as a result of compensation or reorganization, would come from the observation of semantic errors in a patient in the subacute or chronic phase who did not produce any such errors acutely. Research to identify this pattern of oral reading performance is currently underway.

## 5. Conclusion

One of the main sources of evidence for the underlying processes involved in both normal and impaired reading has been the study of acquired dyslexia. However, the vast majority of studies have examined dyslexic reading in the chronic stages of brain injury, and few, if any, have examined patterns of reading found acutely. Reading performance in the acute phase is of interest as it allows the examination of possible mechanisms involved in normal and impaired reading without reorganization of functioning. In addition, reading patterns in these individuals may help to shed light on models of normal and impaired reading, which may predict different patterns of performance at the acute and chronic stages. The current study aimed to be the first step in furthering in our knowledge regarding reading performance in the acute stages of brain injury. Many similarities were found in the reading performance of individuals after acute brain damage and those more chronic patients reported in previous literature. However, despite these similarities, some interesting differences in the patterns of errors produced were observed. The task now remains to confirm the existences of these differences, and to examine what significance, if any, these differences have in relation to current models of oral reading.

## Disclosure

The authors have no relationships to disclose.

## References

[1] A. Colangelo and L. Buchanan, Semantic ambiguity and the failure of inhibition hypothesis as an explanation for reading errors in deep dyslexia, *Brain and Cognition* **57** (2005), 39–42.
[2] A. Colangelo and L. Buchanan, Localizing damage in the functional architecture: The distinction between implicit and explicit processing in deep dyslexia, *Journal of Neurolinguistics* **20** (2007), 111–144.
[3] A.A. Beaton and N.W. Davies, Semantic errors in deep dyslexia: Does orthographic depth matter? *Cognitive Neuropsychology* **24** (2007), 312–323.

[4] A.E. Hillis and A. Caramazza, Mechanisms for accessing lexical representations for output: Evidence from a category-specific semantic deficit, *Brain and Language* **40** (1991), 106–144.
[5] A.E. Hillis and A. Caramazza, The reading process and its disorders, in: *Cognitive Neuropsychology in Clinical Practice*, D. Margolin, ed., Oxford University Press, New York, 1992, pp. 229–261.
[6] A.M. Woollams, M.A. Lambon Ralph, D.C. Plaut and K. Patterson, SD-squared: On the association between semantic dementia and surface dyslexia, *Psychological Review* **114** (2007), 316–339.
[7] C. Perry, J.C. Ziegler and M. Zorzi, Nested incremental modelling in the development of computational theories: The CDP+ model of reading aloud, *Psychological Review* **114** (2007), 273–315.
[8] C.J. Price, C.J. Moore and R.S.J. Frackowiak, The effect of varying stimulus rate and duration on brain activity during reading, *NeuroImage* **3** (1996), 40–52.
[9] C.J. Price, D. Howard, K. Patterson, E.A. Warburton, K.J. Friston and S.J. Frackowiak, A functional neuroimaging description of two deep dyslexic patients, *Journal of Cognitive Neuroscience* **10** (1998), 303–315.
[10] C.J. Price, M.L. Gorno-Tempini, K.S. Graham, N. Biggio, A. Mechelli, K. Patterson and U. Noppeney, Normal and pathological reading: Converging data from lesion and imaging studies, *NeuroImage* **20** (2003), S30–S41.
[11] D. Bub, Alexia and related reading disorders, *Neurologic Clinics of North America* **21** (2003), 549–568.
[12] D. Chialant and A. Caramazza, Perceptual and lexical factors in a case of letter-by-letter reading, *Cognitive Neuropsychology* **15** (1998), 187–201.
[13] D. Saur, R. Lange, A. Baumgaertner, V. Schraknepper, K. Willmes, M. Rijntjes and C. Weiller, Dynamics of language reorganisation after stroke, *Brain* **129** (2006), 1371–1384.
[14] D.C. Plaut, Structure and function in the lexical system: Insights from distributed models of word reading and lexical decision, *Language and Cognitive Processes* **12** (1997), 765–805.
[15] D.C. Plaut, A connectionist approach to word reading and acquired dyslexia: Extension to sequential processing, *Cognitive Science* **23** (1999), 543–568.
[16] D.C. Plaut, J.L. McClelland, M.S. Seidenberg and K. Patterson, Understanding normal and impaired word reading: Computational principles in quasi-regular domains, *Psychological Review* **103** (1996), 56–115.
[17] E. Jefferies, K. Sage and M.A. Lambon Ralph, Do deep dyslexia, dysphasia, and dysgraphia share a common phonological impairment? *Neuropsychologia* **45** (2007), 1553–1570.
[18] E.B. Marsh and A.E. Hillis, Cognitive and neural mechanisms underlying reading and naming: Evidence from letter-by-letter reading and optic aphasia, *Neurocase* **11** (2005), 325–337.
[19] E.L. Breese and A.E. Hillis, Auditory comprehension: Is multiple choice really good enough, *Brain and Language* **89** (2004), 3–8.
[20] F. Michel, M.-A. Henaff and J. Intriligator, Two different readers in the same brain after a posterior callosal lesion, *NeuroReport* **7** (1996), 786–788.
[21] G. Glosser and R. Friedman, The continuum of deep/phonological dyslexia, *Cortex* **25** (1990), 343–359.
[22] G. Miceli, R. Capasso and A. Caramazza, Sublexical conversion procedures and the interaction of phonological and orthographic lexical forms, *Cognitive Neuropsychology* **16** (1999), 557–572.
[23] G.E. Hinton and T. Shallice, Lesioning an attractor network: Investigations of acquired dyslexia, *Psychological Review* **98** (1991), 74–95.
[24] H. Goodglass, K.C. Lindfield and M.P. Alexander, Semantic capacities of the right hemisphere as seen in two cases of pure word blindness, *Journal of Psycholinguistic Research* **29** (2000), 399–422.
[25] H.B. Coslett, Read but not write "idea": Evidence for a third reading mechanism, *Brain and Language* **40** (1991), 425–443.
[26] H.B. Coslett, Acquired dyslexia, *Seminars in Neurology* **20** (2000), 419–426.
[27] H.B. Coslett and N. Monsul, Reading with the right hemisphere: Evidence from transcranial magnetic stimulation, *Brain and Language* **46** (1994), 198–211.
[28] I.C. Iribarren, G. Jarema and A.R. Lecours, Lexical reading in Spanish: Two cases of phonological dyslexia, *Applied Psycholinguistics* **20** (1999), 407–428.
[29] I.-K. Bjaalid, T. Hoien and I. Lundberg, Dual-route and connectionist models: A step towards a combined model, *Scandinavian Journal of Psychology* **38** (1997), 73–82.
[30] J. Crisp and M.A. Lambon Ralph, Unlocking the nature of the phonological-deep dyslexia continuum: The keys to reading aloud are in phonology and semantics, *Journal of Cognitive Neuroscience* **18** (2006), 348–362.
[31] J. Dickerson and H. Johnson, Sub-types of deep dyslexia: A case study of central deep dyslexia, *Neurocase* **10** (2004), 39–47.
[32] J. Larsen, K. Baynes and D. Swick, Right hemisphere reading mechanisms in a global aphasic patient, *Neuropsychologia* **42** (2004), 1459–1476.
[33] J.G. Snodgrass and M. Vanderwart, A standardized set of 260 pictures: Norms for name agreement, image agreement, familiarity, and visual complexity, *Journal of Experimental Psychology: Human Learning and Memory* **6** (1980), 174–215.
[34] J.M. Rumsey, B. Horwitz, B.C. Donohue, K. Nace, J.M. Maisog and P. Andreason, Phonological and orthographic components of word recognition: A PET-rCBF study, *Brain* **120** (1997), 739–759.
[35] K. Rastle, L.K. Tyler and W. Marslen-Wilson, New evidence for morphological errors in deep dyslexia, *Brain and Language* **97** (2006), 189–199.
[36] K.E. Patterson, What is right with "deep" dyslexic patients? *Brain and Language* **8** (1979), 111–129.
[37] K.E. Patterson, M. Coltheart and J.C. Marshall, *Surface dyslexia*, LEA, London, 1985.
[38] L. Buchanan, N. Hildebrandt and G.E. MacKinnon, Phonological processing of nonwords by a deep dyslexic patient: A rowse is implicitly a rose, *Journal of Neurolinguistics* **8** (1994), 163–181.
[39] L. Buchanan, N. Hildebrandt and G.E. MacKinnon, Phonological processing of nonwords in deep dyslexia: typical and independent? *Journal of Neurolinguistics* **9** (1995), 113–133.
[40] L. Buchanan, S. McEwen, C. Westbury and G. Libben, Semantics and semantic errors: Implicit access to semantic information from words and nonwords in deep dyslexia, *Brain and Language* **84** (2003), 65–83.
[41] L.J. Buxbaum and H.B. Coslett, Deep dyslexic phenomena in a letter-by-letter reader, *Brain and Language* **54** (1996), 136–167.
[42] M. Beeman, R.B. Friedman, J. Grafman, E. Perez, S. Diamond and M.B. Lindsay, Summation priming and coarse semantic coding in the right hemisphere, *Journal of Cognitive Neuroscience* **6** (1994), 26–45.

[43] M. Coltheart, Deep dyslexia is right-hemisphere reading, *Brain and Language* **71** (2000), 299–309.
[44] M. Coltheart, B. Curtis, P. Atkins and M. Haller, Models of reading aloud: Dual-route and parallel-distributed-processing approaches, *Psychological Review* **100** (1993), 589–608.
[45] M. Coltheart and K. Rastle, Serial processing in reading aloud: Evidence for dual-route models of reading, *Journal of Experimental Psychology: Human Perception and Performance* **20** (1994), 1197–1211.
[46] M. Coltheart, K. Rastle, C. Perry, J. Ziegler and R. Langdon, DRC: A dual route cascaded model of visual word recognition and reading aloud, *Psychological Review* **108** (2001), 204–256.
[47] M. Laine, P. Niemi, J. Niemi and P. Koivuselka-Sallinen, Semantic errors in a deep dyslexic, *Brain and Language* **38** (1990), 207–214.
[48] M. Miozzo and A. Caramazza, Varieties of pure alexia: The case of failure to access graphemic representations, *Cognitive Neuropsychology* **15** (1998), 203–238.
[49] M.A. Lambon Ralph and N.L. Graham, Acquired phonological and deep dyslexia, *Neurocase* **6** (2000), 141–178.
[50] M.J. Farah, R.M. Stowe and K.L. Levinson, Phonological dyslexia: Loss of a reading-specific component of the cognitive architecture? *Cognitive Neuropsychology* **13** (1996), 849–868.
[51] M.W. Harm and M.S. Seidenberg, Phonology, reading acquisition, and dyslexia: Insights from connectionist models, *Psychological Review* **106** (1999), 491–528.
[52] M.W. Harm and M.S. Seidenberg, Computing the meaning of words in reading: Cooperative division of labour between visual and phonological processes, *Psychological Review* **111** (2004), 662–720.
[53] N. Hildebrandt, The Reicher-Wheeler effect and models of deep and phonological dyslexia, *Journal of Neurolinguistics* **8** (1994), 1–18.
[54] P.K. Newton and C. Barry, Concreteness effects in word production but not word comprehension in deep dyslexia, *Cognitive Neuropsychology* **14** (1997), 481–509.
[55] R. de Bleser, J. Faiss and M. Schwarz, Rapid recovery of aphasia and deep dyslexia after cerebrovascular left-hemisphere damage in childhood, *Journal of Neurolinguistics* **9** (1995), 9–22.
[56] R.B. Friedman, Recovery from deep alexia to phonological alexia: Points on a continuum, *Brain and Language* **52** (1996), 114–128.
[57] R.B. Friedman and M.B. Perlman, On the underlying causes of semantic paralexias in a patient with deep dyslexia, *Neuropsychologia* **20** (1982), 559–568.
[58] R.S. Berndt, A.N. Haendiges, C.C. Mitchum and S.C. Wayland, An investigation of nonlexical reading impairments, *Cognitive Neuropsychology* **13** (1996), 763–801.
[59] S. Gerhand, Routes to reading: A report of a non-semantic reader with equivalent performance on regular and exception words, *Neuropsychologia* **39** (2001), 1473–1484.
[60] S. Gerhand and C. Barry, When does a deep dyslexic make a semantic error? The roles of age-of-acquisition, concreteness, and frequency, *Brain and Language* **74** (2000), 26–27.
[61] S.Y. Bookheimer, T.A. Zeffiro, T. Blaxton, W. Gaillard and W. Theodore, Regional cerebral blood flow during object naming and word reading, *Human Brain Mapping* **3** (1995), 93–106.
[62] S.Z. Rapcsak, M.L. Henry, S.L. Teague, S.D. Carnahan and P.M. Beeson, Do dual-route models accurately predict reading and spelling performance in individuals with acquired alexia and agraphia? *Neuropsychologia* **45** (2007), 2519–2524.
